# TCP post-radioembolization and TCP post-EBRT in HCC are similar and can be predicted using the in vitro radiosensitivity

**DOI:** 10.1186/s13550-022-00911-0

**Published:** 2022-07-08

**Authors:** Philippe d’Abadie, Stephan Walrand, Michel Hesse, Ivan Borbath, Renaud Lhommel, François Jamar

**Affiliations:** 1 Department of Nuclear Medicine, Cliniques Universitaires Saint Luc, Institut Roi Albert II, 10, avenue Hippocrate, 1200 Brussels, Belgium; 2 Department of Medical Oncology, CIiniques Universitaires Saint Luc, Institut Roi Albert II, Brussels, Belgium

**Keywords:** HCC, TCP, EUD, PET, FDG, Acetate

## Abstract

**Background:**

Tumor equivalent uniform dose (EUD) is proposed as a predictor of patient outcome after liver radioembolization (RE) of hepatocellular carcinoma (HCC) and can be evaluated with ^90^Y-TOF-PET. The aim is to evaluate the correlation between PET-based tumors EUD and the clinical response evaluated with dual molecular tracer (^11^C-acetate and ^18^F-FDG) PET/CT post-RE.

**Methods:**

34 HCC tumors in 22 patients were prospectively evaluated. The metabolic response was characterized by the total lesion metabolism variation (ΔTLM) between baseline and follow-up. This response allowed to compute a tumor control probability (TCP) as a function of the tumor EUD.

**Results:**

The absorbed dose response correlation was highly significant (*R* = 0.72, *P* < 0.001). With an absorbed dose threshold of 40 Gy, the metabolic response was strongly different in both groups (median response 35% versus 100%, *P* < 0.001). Post-RE TCP as a function of the EUD was very similar to that observed in external beam radiation therapy (EBRT), with TCP values equal to 0.5 and 0.95 for a EUD of 51 Gy and 100 Gy, respectively. The TCP was perfectly predicted by the Poisson model assuming an inter tumor radiosensitivity variation of 30% around the HCC cell in vitro value.

**Conclusions:**

EUD-based ^90^Y TOF-PET/CT predicts the metabolic response post-RE in HCC assessed using dual molecular PET tracers and provides a similar TCP curve to that observed in EBRT. In vivo and in vitro HCC radiosensitivities are similar. Both TCPs show that a EUD of 100 Gy is needed to control HCC for the three devices (resin spheres, glass spheres, EBRT). Observed absorbed doses achieving this 100 Gy-EUD ranged from 190 to 1800 Gy!

**Supplementary Information:**

The online version contains supplementary material available at 10.1186/s13550-022-00911-0.

## Background

Liver radioembolization (RE) is part of the treatment strategy of hepatocellular carcinoma (HCC) [1] and aims to deliver high efficient absorbed doses to tumors [2]. The tumor-absorbed dose is a predictive factor of the treatment’s effectiveness [3]. Comparing the different types of radiation therapies, the D_50_ giving a 50% tumor control probability (TCP) for glass and resin microspheres is about 250 Gy [4] and 60 Gy [5, 6], respectively, and about 53 Gy for [7] external beam radiation therapy (EBRT). Centimeter scale heterogeneities in the activity distribution in RE explain these differences [8]. Such heterogeneity is sufficiently revealed by time of flight (TOF)-PET ^90^Y imaging [8–10] to allow reunification of the efficacy threshold doses observed in RE (resin or glass spheres) and in EBRT using the EUD [11]. Previous radiobiological models developed in RE for HCC demonstrated a correlation between the tumor-absorbed dose and the clinical response assessed with conventional imaging [4–6]. In colorectal liver metastases, a similar correlation was demonstrated using the metabolic response assessed with ^18^F-FDG PET/CT [12, 13].

Functional imaging (PET/CT) is currently not recommended for the management of HCC, especially because ^18^F-FDG PET/CT has a low diagnostic sensitivity, with a definite uptake in less than 50–65% of the patients [14]. ^11^C-Acetate PET/CT is more sensitive for HCC detection, with an uptake reached in 85% of patients [15].

^11^C-Acetate enters the Krebs cycle as a substrate for β-oxidation in fatty acid and cholesterol synthesis which is likely the explanation of its uptake by differentiated HCC [15]. On the other hand, the high level of glucose-6-phosphatase in differentiated HCC leads to the release of ^18^FDG while poorly differentiated HCC, which has a low abundance of this enzyme, tends accumulate ^18^F-FDG [14, 15]. As a result, performing a dual molecular tracer PET/CT acquisition (^11^C-acetate and ^18^F-FDG) was able to improve this sensitivity up to 98% [16].

This study aims to evaluate the ability of ^90^Y TOF-PET/CT-based EUD to predict metabolic response assessed with dual molecular PET tracer.

## Methods

### Patients

A total of 23 HCC patients, diagnosed with contrast enhanced MRI or CT scan, referred to our department for RE, were prospectively enrolled in this study after their informed consent and approval by the local ethics committee (2015/01OCT/522).

Each patient was evaluated with dual isotope (^11^C-acetate and ^18^F-FDG) PET/CT at baseline and at follow-up (FU) 2 and 4 months after RE. This study protocol was proposed to each patient referred for RE by our multidisciplinary oncology team between 2015 and 2019. No exclusion criteria were applied.

Four patients received a second treatment after an interval of 5 months and were evaluated again. Four patients had multifocal tumors (2 to 6), while the majority had only one tumor.

A total of 34 tumors demonstrated a metabolic uptake, 80% were positive with acetate PET/CT, 52% with FDG PET/CT and 40% with both imaging. In one patient, the tumor uptake was not significant with either PET tracers and the metabolic evaluation was not possible, resulting in 22 studied patients. No other exclusion factor was applied.

The patients characteristics are summarized in Table 1.

**Table 1:** Patients characteristics

Characteristics	Data
Age (years)	73 (68–79)
Sex	Male: 14 (64%)
	Female: 8 (34%)
BCLC staging	A: 8 (36%)
	B: 8 (36%)
	C: 6 (28%)
Tumor volume (ml)	36 (11–97)

Continuous variables described with median and 95% confidence interval displayed between parentheses

*BCLC* Barcelona Clinic Liver Cancer

### Treatment

RE was performed according to standard recommendations [17–19]. Resin microspheres (Sir-Spheres®, Sirtex Medical Ltd., Sydney, Australia) and glass microspheres from 1 up to 4 days post-calibration (Therasphere®, Boston Scientific, Boston, MA) were used in 17 and 9 treatments, respectively. No systematic criteria have driven the choice of the therapy. Nevertheless, patients with small and solitary tumors were treated mostly at a segmental level using glass microspheres to concentrate a high activity in a small volume.

A 40-min 2-bed positions ^90^Y TOF-PET scan (Gemini TF64 Philips Medical Systems, Cleveland, OH) was acquired within 4 h following the RE. Reconstruction was performed with the 3D line of response (LOR)–TOF blob-based OSEM algorithm from Philips with 2 iterations times 33 subsets and a 4 × 4 mm^3^ voxel size. ^90^Y activity distribution was transformed into a 3D-map of absorbed doses using a previously validated method [20]. In summary, voxel counts were converted to absorbed dose distribution by convolving the ^90^Y activity distribution with a dose point kernel, after spatial resolution recovery.

### Tumor response assessment

Whole-body TOF-PET/CT acquisition (Gemini TF 64, Philips Medical Systems, Cleveland, OH) was acquired 20 min after intravenous injection of ^11^C-acetate (370–540 MBq, half-life: 20 min). 30 min after this first acquisition, ^18^F-FDG was injected (280–310 MBq) and images were acquired after an incorporation of 60 min, i.e., about 130 min post ^11^C-acetate injection, resulting in less than 5 MBq of ^11^C. Acquisitions were performed at baseline and during FU at 2 and 4 months after treatment.

Tumor contours were automatically defined with acetate or FDG PET/CT using an isocontour method (41% SUVmax threshold) performed with the MIM software (V7.1, MIM Software Inc., Cleveland, OH).

For each patient, the tracer, i.e., ^11^C-acetate or ^18^F-FDG, giving the highest tumor to normal liver uptake ratio on the baseline PET was selected to assess the tumor response metabolism.

For each tumor and each time point, the total lesion metabolism (TLM) was defined as:

1 $${\text{TLM}} = {\text{SUV}}_{{{\text{mean}}}} { } \times {\text{Volume}}$$

The ΔTLM between baseline (B) and FU defined the metabolic response:

2 $$\Delta T{\text{LM }}\left( {{\% }} \right) = \frac{{{\text{TLM}}_{{\text{B}}} - {\text{TLM}}_{{{\text{FU}}}} }}{{{\text{TLM}}_{{\text{B}}} }} \cdot 100$$

The best metabolic response between the baseline and either 2 or 4 month was considered (corresponding to the maximal effects of radiations).

No TLM threshold are established to classify the metabolic response of tumors. However, regarding EUD, an absorbed dose threshold of 40 Gy was previously defined as an efficacy threshold in HCC [11] and corresponded also to the efficacy threshold in EBRT [21]. Therefore, this threshold was applied for comparing the metabolic responses.

### Tumor dosimetry

Tumor contours were matched with the 3D map of absorbed doses, as previously defined, using a rigid co-registration with the MIM software.

Equivalent uniform dose (EUD) was calculated according to the Jones and Hoban formalism [22]:

3 $${\text{EUD}} = - \frac{1}{{\alpha^{*} }}\ln \left( {\frac{{\mathop \sum \nolimits_{i} e^{{ - \alpha^{*} D_{i} }} }}{{N_{v} }}} \right)$$

where α* is the apparent HCC radiosensitivity, *N*_*v*_ the number of voxels in the tumor and *D*_*i*_ the absorbed dose in tumor voxel *i*. The terminology apparent radiosensitivity was introduced by Chiesa et al. [4]. This value depends on the spatial resolution of the dose distribution assessment as shown in [10]. In a previous ^90^Y TOF-PET-based EUD analyses, the apparent α* coefficient was estimated to be 0.038 Gy^−1^ [11], i.e., about tenfold lower than the intrinsic in vitro HCC 0.40 Gy^−1^ radiosensitivity *α* [23].

### TCP assessment

#### The TCP was determined in consecutive EUD bins of 20 Gy

The metabolic response threshold (MRT) and the apparent radiosensitivity α*, common to resin and glass sphere, were simultaneously fitted in order to get the best agreement with the TCP observed in EBRT after 6 months, i.e., using ΔTLM > MRT (Eq. 2) as responding criteria in bin EUD (Eq. 3) [7]. Note that this implicitly means that the survival fraction is given by $e^{{ - \alpha \;{\text{EUD}}}}$ and not by $e^{{ - \alpha^{*} \;{\text{EUD}}}}$ (we will later discus the rationale of using two different radiosensitivity concepts in the same dose–response modeling).

The tumor response in this EBRT study was assessed using radiographic RECIST v 1.1 or a < 20% AFP decrease, depending on data availability. The end point was 6 months post-treatment. The TCP was fitted using a logistic function which did not provide a radiosensitivity assessment.

### Tumor cells radiosensitivity

According to the Poisson law, the probability to observe n_s_ surviving cells after an irradiation EUD of a cell colony is given by:

4 $$P\left( n \right) = \frac{{\left( {N_{s} \left( {{\text{EUD}}} \right)} \right)^{{n_{s} }} }}{{n_{s} !}} e^{{ - N_{s} \left( {{\text{EUD}}} \right)}}$$

where $N_{s} \left( {{\text{EUD}}} \right)$ is the mean observed number of surviving cells after many identical irradiations of identical cell colonies, which for clonogenic cells colony is given by:

5 $$N_{s} \left( {{\text{EUD}}} \right) = N_{0} e^{{ - \alpha \;{\text{ EUD}}}}$$

where *α* is the intrinsic cell radiosensitivity and *N*_0_ the number of living cells before irradiation.

Assuming that a tumor is controlled only if all the cells are killed, the TCP is then given by *P*(0) [24], i.e.:

6 $${\text{TCP}}\left( {{\text{EUD}}} \right) = P\left( 0 \right) = e^{{ - N_{0} e^{{ - \alpha \;{\text{EUD}}}} }}$$

In clinical research, the radiosensitivity of the tumors used to build the TCP can be different from one patient to another, according to the HCC stage, to the patient hematocrit [25, 26]. Assuming that the radiosensitivity distribution in the tumor population is normal, we can approximate the TCP by:

7 $$TCP\left( {EUD} \right) = \frac{1}{{\sqrt {2\pi } \sigma }}\smallint e^{{ - \frac{{\left( {\alpha^{\prime} - \alpha } \right)^{2} }}{{2 \sigma^{2} }}}} e^{{ - N e^{{ - \alpha^{\prime}EUD}} }} d\alpha^{\prime}$$

where *σ* is the standard deviation of the radio-sensitivity *α* and <*N*> the number of living tumors cells in average before irradiation.

### TCP error bars

Ideally, for each EUD bin the tumor control assessment should be a Bernoulli trial, i.e., a random experiment having two possible outcomes with a constant probability of control being TCP(EUD). In such trial, the likelihood follows the binomial distribution:

8 $$L(n|N:{\text{TCP}}\left( {{\text{EUD}}} \right)) = N! \frac{{ {\text{TCP}}^{n} }}{n!} \frac{{ \left( {1 - {\text{TCP}}} \right)^{N - n} }}{{\left( {N - n} \right)!}}$$

where *n* is the number of responding tumors within the *N* tumors investigated and having received the dose EUD. A simple partial derivative of Eq. 8 versus TCP shows that the TCP maximizing the likelihood is:

9 $${\text{TCP}}_{L\max } = \frac{n}{N}$$

the maximal likelihood value being:

10 $$L_{\max } = N! \frac{{n^{n} }}{{N^{n } n!}} \frac{{ \left( {1 - n/N} \right)^{N - n} }}{{\left( {N - n} \right)!}}$$

Note that the binomial distribution is asymmetric. We will represent the error bars in a conventional way, i.e., corresponding to left and right widths at half maximum divided by 2.35 (we will later discuss this choice). The equivalent lower and upper standard deviations are thus:

11 $$\sigma^{l,u} = \frac{2}{2.35} \left| {{\text{TCP}}_{L\max } - {\text{TCP}}_{L\max /2}^{l,u} } \right|$$

where $TCP_{L\max /2}^{l,u}$ are the two TCP values giving $L_{\max } / 2$ on left and right side of ${\text{TCP}}_{L\max }$. These TCPs are the solutions of:

12 $$\left( \frac{n}{N} \right)^{n} \left( {1 - \frac{n}{N}} \right)^{N - n} = 2 {\text{TCP}}^{n} \left( {1 - {\text{TCP}}} \right)^{N - n}$$

Equation 12 is a *N*-order equation and for *N* > 4 the solutions are not analytics and can only be numerically computed, excepted in the special case *n* = *N*/2 where the Eq. 12 reduces to a second order one and provides a symmetrical standard deviation:

13 $$\sigma = \frac{{ \sqrt { 1 - 2^{1/n} } }}{2.35}$$

Equation 13 clearly shows that σ → 0 when n → ∞.

### Statistical analysis

A Mann–Whitney *U* test was used to compare the differences in groups. The correlation was analyzed using a Spearman coefficient (R).

Analyses were conducted with Prism software (version 7.0, GraphPad Software, La Jolla, Ca).

## Results

**Fig. 1:**
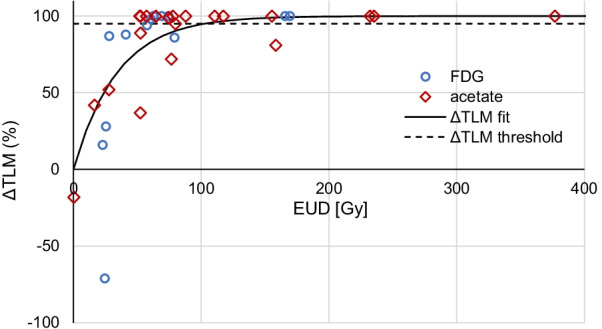
ΔTLM as a function of the EUD obtained from post-RE ^90^Y TOF-PET. Blue circles: measured on ^11^C-acetate PET. Red diamonds: measured on ^18^F-FDG PET. Dashed line: ΔTLM threshold used to discriminate responding and not responding tumor in the TCP computation

The metabolic response correlated well with EUD (Fig. 1): the larger the EUD the higher the metabolic response. This EUD response correlation was highly significant according to the Spearman coefficient mixing both tracers (*R* = 0.72, *P* < 0.001) and similar when splitting the populations, i.e., (*R* = 0.81 for FDG and *R* = 0.64 for acetate). Moreover, the metabolic response to radiations was very similar for tumors positive with FDG PET/CT and with acetate PET/CT (Fig. 1). The metabolic response was significantly higher in tumors receiving more than 40 Gy (median ΔTLM: 1.0) compared to tumors receiving a lower EUD (median ΔTLM: 0.35, *P* < 0.001).

**Fig. 2:**
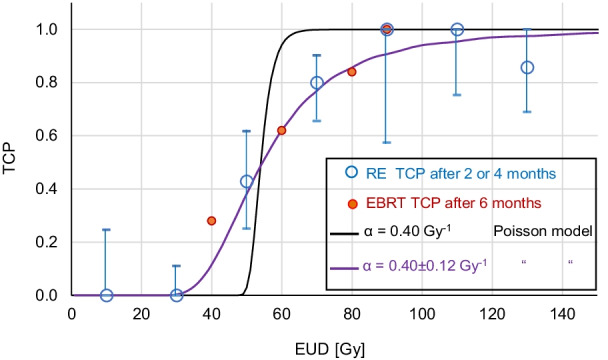
TCP as a function of the EUD. Blues circles: this RE study. Red bullets: External beam radiation therapy (EBRT) derived from Lausch et al. [7]. Solid lines: Poisson TCP models using the invitro radiosensitivity for clonogenic cells colony (black) and for tumors population (violet)

Figure 2 shows the agreement between the TCP observed in RE after 2 or 4 months and in EBRT after 6 months, the fitted apparent radiosensitivity for the EUD computation was α* = 0.035 Gy^−1^ quite close to the previous study (0.038 Gy^−1^ in [11]). The clonogenic Poisson model (Eq. 6) using the invitro radiosensitivity (0.40 Gy^−1^ [23]) and the mean tumor cells $N = 2.5 \times 10^{9}$ corresponding to the measured tumor volume (36 ml Table 1) rightly predicted the D_50_, but with exhibiting a step shape. A small radiosensitivity standard deviation of 0.12 Gy^−1^ between tumors gave the right TCP shape (Eq. 7).

An excel file including anonymized tumor dose distributions together with a sheet allowing the TCP computation and fitting is available at the Mendeley repository site: https://doi.org/10.17632/cp2vp4cjcs.1

## Discussion

### Findings

This study confirms even on HCC, seldom investigated with metabolic tracers, the strong correlation between reduction of tumor metabolism and EUD. This had been previously observed in colorectal cancer liver metastases that are more diffusely investigated with FDG [12].

A novelty of the present observation is that this correlation holds true when mixing acetate and FDG data for the tumor metabolism assessment. This makes sense considering the fact that reduction of acetate or FDG uptake mainly results from the reduction of tumor living cells number. Indeed, proteins which constitute the metabolic engines are hugely radioresistant (Fig. 1a in [27]).

Beyond this dose–response confirmation, the main finding of the study is that the observed TCP as a function of the EUD in RE is very similar to that of EBRT. We already demonstrated [11] that ^90^Y TOF-PET-based EUD provided the same threshold to discriminate “responding” and “non-responding” patients in glass RE, resin RE and EBRT, i.e., 40 Gy. This is the major benefit versus using mean absorbed that can also provide similar dose correlation when tuning an efficacy factor to the treatment device used [6, 11, 28], i.e., resin spheres, glass spheres or EBRT.

Beside the scientific satisfaction to better understand the therapeutic efficiency of these different treatments, it opens the way to predict the efficiency, and toxicity, of not yet used device [29], e.g., different specific sphere activities obtained by glass spheres decaying, or obtained by using new isotopes, such the recent ^166^Ho.

### EUD assessment and voxel size

With regard to the finite voxel size (4 mm^3^), one can ask whether it is possible to get a tumor with all voxels exhibiting high curative probability voxels dose (i.e., > 80 Gy), while cell subsets in some voxels get low curative probability doses (i.e., < 40 Gy). In the affirmative, current PET imaging could fail to provide a predictive EUD assessment. Amazingly, although that the dose kernel has been shown to quickly decrease by a two orders of magnitude in a 1 mm range [30], this scenario is impossible.

Indeed, the dose *D*_*s*_ (in Gy) at a distance *r*_0_ (in mm) to the edge of a sphere of activity *A* (in kBq) and of radius *R* (in mm) is already accurately modeled by the Russell’s dose kernel [31] when *R* < 1 mm (see Additional file 2: Appendix B for the demonstration):

14 $$D_{s} \left( {r_{0} } \right) = 0.989 A \frac{1}{{\left( {R + r_{0} } \right)^{2} }} \left( {1 - \frac{{R + r_{0} }}{8}} \right)$$

Let us consider 2.5 kBq glass sphere which is the most challenging device in term of sub-voxel dose heterogeneity. The number of spheres needed inside a 4 mm^3^ voxel to get a dose of 80 Gy is 41. Assume the worst scenario in which all the spheres are fully compacted at the voxel center, then the cluster diameter is *R* = 0.061 mm (assuming a rigid sphere compaction factor, i.e., 0.6).

In this scenario, Eq. 14 predicts a huge dose of 27,036 Gy at the cluster contact, and a dose 4.8 Gy at the voxel corners which are 3.4 mm far to the cluster edge. However, as all the tumor voxels are assumed to exhibit curative doses, voxel corners are in fact irradiated by 8 surrounding clusters, located at similar distance, giving 8 × 4.8 = 38.4 Gy, and by 24 surrounding clusters distant of ≈ 6.325 mm giving 24 × 0.53 = 12.7 Gy.

As a result, the lowest cell dose in the voxel is 51 Gy, above the low curative 40 Gy dose. Note that 90% of the minimal dose within a voxel arises from the spheres trapped in the surrounding voxels. This shows that the minimal dose within a 4mm^3^ voxel in a tumor exhibiting no centimetric activity heterogeneities is rather independent to the sphere distribution within the voxel. So, the 4mm^3^ voxel size appears sufficiently small to address most of intra-voxel activity heterogeneity issues.

### EUD assessment and PET FWHM

The ^90^Y dose kernel FWHM is narrower than that of PET systems [32]. A similar concern thus arises: could the PET FWHM hide an activity fluctuation having an amplitude and an extension sufficiently large to produce into a voxel a drop from an apparent curative dose to a real non-curative one. There is no simple theoretical argument to answer this question as 2 mechanisms are used to dump the PET PSF impact: the Richardson–Lucy PET PSF deconvolution and the use of an apparent radiosensitivity α* to get a predictive EUD. This last mechanism is especially difficult to theoretically predict due to the nonlinearity of the Jones–Hoban EUD (Eq. 3).

**Fig. 3:**
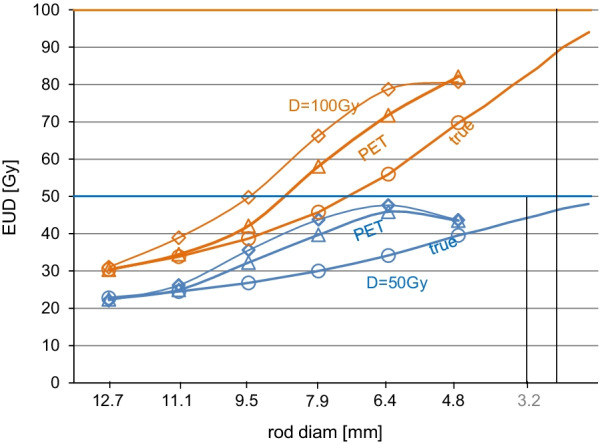
Comparison of the TOF-PET-based EUD for the 6 hot rod sectors of an ultra-deluxe Jaszczak phantom filled with ^90^Y (triangles, diamonds are without and with 6 mm-FWHM filtering, respectively) with the true sectors for a mean sector absorbed dose D of 50 Gy (blue) and 100 Gy (brown). Note that due to the nonlinearity of Eq. 3 the 100 Gy curves shapes are not similar to those of the 50 Gy setup. (reprinted from [10], true EUD computation was extended down to 3.2 mm by the present authors)

However, phantom studies supported that these 2 PSF compensations used together are sufficient to avoid such scenario as shown in Fig. 3: below a 3 mm scale activity pattern distribution, the EUD already reaches 90% of the mean absorbed dose value. In fact, centimetric heterogeneity activity patterns are typically met in sphere radioembolization (see Fig. 1 in [10]). This explains why ^90^Y TOF-PET-based EUD is able to rightly take into account the impact of the heterogeneity of the absorbed dose distribution.

### EUD assessment and tumor delineation

Tumors delineation in dosimetry study is always a major critical step, especially when heterogeneous large tumors are present. Beside the challenge to get an observer independent technique, the key point is in the definition of what is the actual tumor tissue. In dose–response correlation study, only the absorbed dose to the tumor tissue still able to proliferate has to be considered. This justifies our choice to exclude necrotic core using a simple PET uptake iso-contour. This simple method was already successfully used in previous studies [10, 11].

**Fig. 4:**
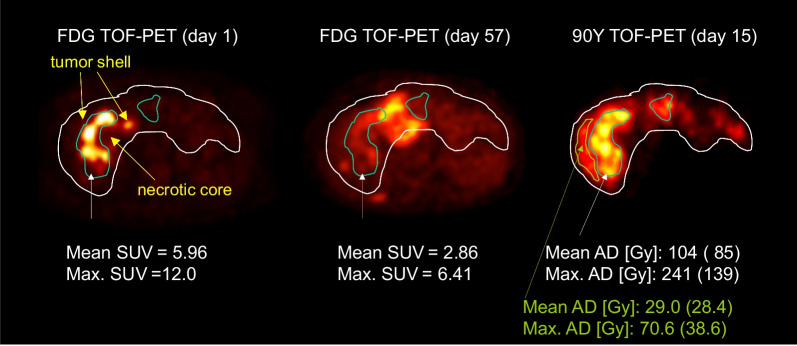
Baseline (day 1) and follow-up (day 57) FDG TOF PET scan of the patient liver compared to the ^90^Y TOF PET raw activity distribution measured after ^90^Y-labeled SIR-Spheres therapy (day 15). A nice tumor response was noted in the region of high absorbed dose (104 Gy), while a tumor progression occurred in the region not targeted by the ^90^Y-labeled SIR-Spheres. Absorbed doses (AD) were computed using the ^90^Y 4-mm voxel S values with (spatial resolution deconvolution) SRD and in parentheses without SRD. (Reprinted from [20] with permission of Springer, yellow notations were added by the authors)

Figure 4 clearly shows a typical response in a large necrotic tumor: the left part of the tumor shell well targeted by the sphere responded, while a small non-targeted region in the right part quickly relapsed in a new necrotic tumor. In the same time, the initial necrotic core, although not targeted, remained stable, i.e. necrotic.

### Radiosensitivity and TCP

In this study, we found an apparent radiosensitivity α* value of 0.035 Gy^−1^ for the computation of EUD. Note that this value not only depends on the imaging spatial resolution, but depends on the spatial resolution of the used dose distribution. When using the mean absorbed dose, this spatial resolution is de facto the tumor diameter, i.e., a few centimeters. This explains why studies using the mean dose [6] found apparent radiosensitivity tenfold lower.

Due to the huge number of cells present in a tumor, the Poisson model gave a step function for the TCP (Fig. 2). This issue has been empirically solved in several studies by fitting together N and α in Eq. 6, which resulted in tenfold lower radiosensitivity and unrealistic tumor cells number ranging from 0.4 [33] to 3.4 [4, 34]. In fact, using the tumor cell number corresponding to the mean tumor volume (36 ml) and a standard deviation of 30% around the in vivo HCC cell radiosensitivity between patients is sufficient to rightly predict the observed TCP shape (Fig. 2).

Note that the nonclonogenicity within a tumor does not change its TCP step shape. Indeed, the TCP of such tumor is obtained by the product of the P(0) (Eq. 6) corresponding to the different clonogenic subsets. This product can be translated into a summation in the exponent which does not change the step shape. The step shape smoothing is the result of compiling together responses of tumors having different radiosensitivities and different volumes in order to build the TCP.

As a result, the TCP assessment is not a true Bernoulli trial which should had required that for a specific EUD all studied tumors would have the same probability to be controlled. Even for a true Bernoulli trial, the methodology choice for accurate confidence estimation is still under debate [35] and is fully unknown in the clinical case described by Eq. 7. It is why we preferred to use the likelihood fwhm/2.35 as an estimator of the potential error.

It could appear amazing that using the mean tumor volume (36 ml) is sufficient to predict the TCP, while there is a tenfold factor between the observed tumor volumes (Table 1). This results from the exponential behavior of the cells surviving fraction: an additive of only 6 Gy to the EUD is sufficient to reduce the survival fraction by this tenfold factor.

We want also to emphasize that the radiosensitivity used in the EUD derivation and the one used in the TCP modeling are two different concepts. An apparent radiosensitivity α* has to be used in the EUD derivation in order to compensate the limited spatial resolution obtained for in vivo dose distribution. After obtaining the right EUD, it is obvious that the surviving tumor cells number is governed by the intrinsic tumor cells radiosensitivity *α*.

The purpose of the study was not to evaluate the efficiency of radioembolization, in which case it should have been suitable to use the recommended mRECIST method. The study purpose was to investigate the rightness of a EUD and of a response assessment methodology, i.e. PET uptake iso-contour based EUD together with metabolic PET response. The rightness of this choice is clearly supported by the fact that the methodology provided similar dose-TCP independently of the treatment devices (resin, glass or EBRT).

Also, note that the Poisson theory shows that TCP is governed by the number of surviving tumor cells. To this regard, metabolic response assessment is a more direct estimation of the number of surviving cells than anatomical image which cannot differentiate between surviving cells and dead cells not yet cleared by the immune system.

The optimal ΔTLM 95% threshold, and not 100%, used to obtain the best agreement results from different effects: errors in the TLM assessment resulting from breathing motion, inflammatory response, variation in other competing tissues metabolism; FU delay too short to allow all the tumor cells to die or in contrary FU delay sufficiently long enough to allow a repopulation of the tumor site by healthy liver tissue which also takes up ^11^C-acetate or ^18^F-FDG.

### Therapeutic considerations

The similarity of the RE and EBRT TCPs consolidates that a EUD of 100 Gy is needed to efficiently control a tumor, i.e., TCP > 0.95. The sigmoid shape of the TCP curve enlightens the importance to reach at least a EUD of 100 Gy in each tumor and emphasize the necessity to optimize the treatment to reach this goal [3, 27, 28]. Note that the absorbed dose D needed to achieve this EUD can vary from 190 to 1800 Gy depending on the absorbed dose distribution heterogeneity (Additional file 1: Appendix A).

The observed RE TCP also explains why the EUD = 40 Gy threshold split the patient survival curves into 2 clear different groups [26]. Indeed, this threshold correspond to the EUD_50_. As a result, one group contains a majority of responding patients and the other one a majority of non-responding patients.

### Limitations

This study has some limitations. First, analyses were performed in a limited number of patients and tumors compared to previous reported studies [4, 6, 12]. Only few tumors received low absorbed doses and demonstrated a poor metabolic response, limiting the accuracy of our radiobiological model for low doses. Second, the follow-up was 4 months at maximum but some tumors could respond later to radiations and hence the effects of radiations could be sometimes underestimated, justifying the choice of the threshold ΔTLM = 95% rather than 100% in the TCP derivation. EUD is based on a single alpha value and it is likely that the radiosensitivity may vary between HCC occurring within different clinical entities. Accordingly, this delayed response may explain why some tumors did not disclose complete metabolic response 4 months after therapy despite a very efficient EUD.

## Conclusions

^90^Y-TOF-PET-based EUD calculated with α* = 0.035 Gy^−1^ is strongly correlated with the metabolic response in HCC assessed by dual molecular tracer PET imaging and is associated with a TCP close to that in EBRT. ^90^Y-TOF-PET-based EUD reunifies absorbed dose levels in RE and EBRT. The use of this parameter must be encouraged in future dosimetry studies for a better understanding of the efficacy threshold absorbed doses in RE. Taking into account the inter tumor radiosensitivity variability predicts the observed TCP using the observed mean tumor cells number and the HCC in vitro radiosensitivity into the Poisson model. A EUD of 100 Gy is needed and sufficient to efficiently control a tumor. The observed absorbed dose D needed to achieve this EUD varied from 190 to 1800 Gy!

## Supplementary Information

**Additional file 1: Appendix A**. Data reporting for each tumor the absorbed dose (D), the equivalent uniform dose (EUD) and the metabolic response (diff TLM)

**Additional file 2: Appendix B**.

## Data Availability

Data generated in the study can be found at the Mendeley repository website at: https://doi.org/10.17632/x68ys6nz5d.1.

## Abbreviations

EBRT: External beam radiation therapy
EUD: Equivalent uniform dose
FU: Follow-up
HCC: Hepatocellular carcinoma
RE: Radioembolization
TCP: Tumor control probability
TLM: Total lesion metabolism
