# Supplementary material for: TCP post-radioembolization and TCP post-EBRT in HCC are similar and can be predicted using the in vitro radiosensitivity
Source: EJNMMI Res. 2022 Jul 8;12:40. doi: 10.1186/s13550-022-00911-0 (PMC9270555; doi:10.1186/s13550-022-00911-0)
Supplement: Supplementary file 1 — Additional file 1: Appendix A. Data reporting for each tumor the absorbed dose (D), the equivalent uniform dose (EUD) and the metabolic response (diff TLM) [file 13550_2022_911_MOESM1_ESM.docx]

**Appendix A**

| pat. | tum. | device | PET | D  [Gy] | EUD  [Gy] | diff TLM  [%] |
| --- | --- | --- | --- | --- | --- | --- |
| 1 | 1 | glass | FDG | 306 | 165 | 100 |
| 2 | 2 | resin | acetate | 531 | 370 | 100 |
| 3 | 3 | glass | FDG | 126 | 72 | 100 |
| 4 | 4 | resin | acetate | 191 | 113 | 100 |
| 4 | 5 | resin | acetate | 380 | 232 | 100 |
| 5 | 6 | resin | acetate | 229 | 76 | 95 |
| 5 | 7 | glass | acetate | 970 | 83 | 100 |
| 6 | 8 | resin | FDG | 86 | 56 | 94 |
| 7 | 9 | resin | acetate | 84 | 51 | 37 |
| 8 | 10 | resin | acetate | 95 | 50 | 100 |
| 9 | 11 | resin | FDG | 51 | 27 | 87 |
| 10 | 12 | glass | FDG | 186 | 66 | 100 |
| 11 | 13 | glass | FDG | 146 | 79 | 86 |
| 12 | 14 | glass | acetate | 1811 | 146 | 100 |
| 12 | 15 | glass | acetate | 308 | 154 | 81 |
| 13 | 16 | glass | acetate | 692 | 220 | 100 |
| 14 | 17 | glass | acetate | 128 | 72 | 99 |
| 15 | 18 | resin | FDG | 74 | 22 | 16 |
| 15 | 19 | resin | FDG | 93 | 23 | -71 |
| 15 | 20 | resin | FDG | 317 | 161 | 100 |
| 16 | 21 | glass | acetate | 87 | 51 | 89 |
| 17 | 22 | resin | FDG | 100 | 39 | 88 |
| 17 | 23 | resin | FDG | 179 | 58 | 98 |
| 18 | 24 | resin | acetate | 701 | 105 | 100 |
| 19 | 25 | resin | acetate | 102 | 55 | 100 |
| 19 | 26 | resin | acetate | 111 | 75 | 72 |
| 20 | 27 | resin | acetate | 121 | 62 | 100 |
| 21 | 28 | resin | FDG | 107 | 61 | 100 |
| 22 | 29 | resin | acetate | 28 | 16 | 42 |
| 22 | 30 | resin | acetate | 45 | 27 | 52 |
| 22 | 31 | resin | acetate | 91 | 51 | 100 |
| 22 | 32 | resin | acetate | 100 | 77 | 100 |
| 22 | 33 | resin | acetate | 0 | 0 | -18 |
| 22 | 34 | resin | FDG | 43 | 25 | 28 |
