# Supplementary material for: TCP post-radioembolization and TCP post-EBRT in HCC are similar and can be predicted using the in vitro radiosensitivity
Source: EJNMMI Res. 2022 Jul 8;12:40. doi: 10.1186/s13550-022-00911-0 (PMC9270555; doi:10.1186/s13550-022-00911-0)
Supplement: Supplementary file 2 — Additional file 2: Appendix B. [file 13550_2022_911_MOESM2_ESM.docx]

**Appendix B:**

In radioembolization dosimetry, the ^90^Y point source dose kernel is often modelled by the Russell’s equation (1):

$D\left( r \right)=0.989 A \frac{1-r/8}{r^{2}}$ *(1)*

where D is the dose in Gy, A the ^90^Y activity in kBq, and r the radial distance in millimetres.

Figure 1: representation of the integration geometry.

Let’s compute the dose D_s_(r_0_), where r_0_ is the distance between the point 0 and the edge of the ^90^Y loaded sphere of radius R and of activity A, for $r \in[0,2R]$ we have:

$\cos\theta_{max}= \frac{r_{0}+r}{\sqrt{2r\left( R+r_{0} \right)+r_{0}^{2}}}$ *(2)*

the $D_{s}(r_{0})$ is given by:

$D_{s}(r_{0})= 0.989 \frac{A}{\frac{4}{3}\pi R^{3}} \int_{0}^{2\pi} d\varphi\int_{0}^{2R} dr \int_{\frac{r_{0}+r}{\sqrt{2r\left( R+r_{0} \right)+r_{0}^{2}}}}^{1} \left( 1-\frac{r_{0}+r}{8 cos\theta} \right) dcos\theta$ *(3)*

note that the denominator of eq. 1 has been simplified with the radial dependence of the spherical coordinate Jacobian which clears the singularity.

Posing:

$a=2\pi0.989 \frac{A}{\frac{4}{3}\pi} = 0.989 \frac{3}{2} A$ (4)

we get:

$D_{s}(r_{0})= \frac{a}{R^{3}} \int_{0}^{2R} dr \left( 1-\frac{r_{0}+r}{\sqrt{2r\left( R+r_{0} \right)+r_{0}^{2}}} + \frac{r_{0}+r}{8} ln(\frac{r_{0}+r}{\sqrt{2r\left( R+r_{0} \right)+r_{0}^{2}}}) \right)$ *(5)*

This integration is analytical and give ( https://www.integral-calculator.com/ ):

$D_{s}(r_{0})= 0.989 A \frac{1}{\left( r_{0}+R \right)^{2}}\left( 1 + \frac{3}{128} \frac{r_{0}^{2} \left( r_{0}+2R \right)^{2}\ln\left( \frac{r_{0}+2R}{r_{0}} \right)-2Rr_{0}^{3}-6R^{2}r_{0}^{2}-8R^{3}r_{0}-4R^{4}}{R^{3}} \right)$ (6)

Note that $\lim_{r_{0}\to0} r_{0}^{2} \ln\left( \frac{r_{0}+2R}{r_{0}} \right) = 0$.

Thus:

$D_{s}(0)= 0.989 A \frac{1}{R^{2}}\left( 1 - \frac{3}{32} R \right)$ (7)

However, the numerical handling of the right term is challenging: when r_0_ > 2 R, i.e. about 0.03 mm for radioembolization spheres, the logarithm has to be expended in Taylor series to keep an accurate numerical result.

This expansion cancels all the terms with power of R lower than 3 and gives:

$D_{s}(r_{0})= 0.989 A \frac{1}{\left( r_{0}+R \right)^{2}}\left( 1 -\frac{\left( r_{0}+ R \right)}{8} + \frac{7}{40}\frac{R^{2}}{r_{0}} + \frac{13}{40}\frac{R^{3}}{r_{0}^{2}} + \vartheta\left( \frac{R^{4}}{r_{0}^{3}} \right) \right)$ (8)

Applicable with 1% accuracy when $\frac{13}{40} \frac{R^{3}}{r_{0}^{2}}< {10}^{-2}$, i.e., for r_0_ > 0.01 mm for radioembolization sphere where R < 1mm.

Eq. 7 and 8 show that for radioembolization sphere (R << 1mm), outside the sphere the dose kernel is already almost equal to the Russell’s one.

**References:**

1. Russell J, Carden J, Herron H. Dosimetry calculations for yttrium-90 used in the treatment of liver cancer. Endocurietherapy/Hyperthermia Oncology. 1988;4(171-1):86.
